# Programming‐Assisted Imaging of Cellular Nitric Oxide Efflux Gradients and Directionality via Carbon Nanotube Sensors

**DOI:** 10.1002/smsc.202400493

**Published:** 2025-02-04

**Authors:** Ivon Acosta Ramirez, Sruti Das Choudhury, Carley Conover, Omer Sadak, Nicole M. Iverson

**Affiliations:** ^1^ Department of Biological Systems Engineering, College of Agricultural Sciences and Natural Resources University of Nebraska‐Lincoln Lincoln Nebraska 68504 USA; ^2^ School of Natural Resources, College of Agricultural Sciences and Natural Resources University of Nebraska‐Lincoln Lincoln Nebraska 68504 USA

**Keywords:** biosensor, cell communication, chemical signaling, extracellular analytes, nanoarray, nitric oxide, single‐walled carbon nanotubes

## Abstract

Cell communication via chemical signaling depends on spatial and temporal concentration changes. Nitric oxide (NO), a gaseous signaling molecule, is critical in physiological and pathological processes. However, current NO sensing methods lack the spatiotemporal resolution necessary to study subcellular NO efflux. This study introduces an innovative sensory platform utilizing single‐walled carbon nanotubes (SWNT) as an optical transducer for the spatial and temporal detection of extracellular NO. The platform quantifies NO diffusion gradients produced by human (THP‐1) and murine (RAW 264.7) macrophage cells. The uniform fluorescence distribution of the nanoarray enables precise analysis of NO efflux directionality, both under and surrounding the cell. It is demonstrated that cellular adhesion to the surface of the sensory platform does not affect its fluorescence functionality or sensing response rate. By combining the platform's high spatiotemporal resolution with the advanced analysis methods, the SWNT sensor platform offers a robust tool for studying extracellular NO dynamics within the cellular microenvironment. This work lays the foundation for advanced diagnostic and therapeutic tools elucidating NO cellular communication analysis.

## Graphical abstract

The single‐walled carbon nanotube platform provides uniform fluorescence distribution, enabling pixel‐level resolution of nitric oxide (NO) efflux gradients across multiple cell types. This novel approach facilitates the determination of directionality and spatial variations in extracellular NO release beneath and surrounding cells, offering new insights into cell communication dynamics through programming‐based pixel analysis.

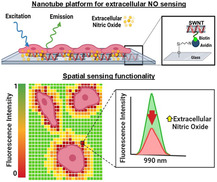

## 1 Introduction

Cell communication, receiving, processing, and transmitting signals between cells and the environment to ensure proper function, can occur directly through physical channels or indirectly via changing concentration gradients of signaling molecules in space and time.^[^ ^1^, ^2^, ^3^ ^]^ Different cellular functional outcomes are regulated by the collective signaling processes within the cell microenvironment.^[^ ^2^ ^]^ For example, through intercellular communication mechanisms, apoptotic cells express proliferative signals to neighboring cells, thereby promoting chemoresistance or treatment resistance.^[^ ^2^ ^]^ Incorporating cell communication dynamics into in vitro diagnostic models can lead to treatment outcomes that are more closely aligned with physiological scenarios.^[^ ^4^, ^5^ ^]^

Among the different molecules involved in cell communication, nitric oxide (NO) is an intra‐ and intercellular signaling molecule involved in different physiological and pathological processes.^[^ ^4^, ^6^ ^]^ NO's small size and gaseous nature enable its diffusion through cell membranes and intercellular compartments to initiate signaling cascades in neighboring cells.^[^ ^7^, ^8^, ^9^ ^]^ Although progress has been made in studying intracellular NO, little is known about the role of NO after being released by cells.^[^ ^10^, ^11^, ^12^, ^13^ ^]^

Quantification of NO in biological systems is challenging due to its high reactivity and short half‐life, which vary from milliseconds in blood to seconds in aqueous solutions.^[^ ^11^, ^14^, ^15^ ^]^ To combat this problem, many researchers measure stable precursor molecules or decomposition products such as nitric oxide synthases (NOS)^[^ ^16^ ^]^ or nitrite (NO_2_^−^) instead of trying to measure NO directly.^[^ ^17^, ^18^ ^]^ Unfortunately, quantification through these methods is also difficult since NO can have multiple degradation pathways that depend on the availability of cofactors in the microenvironment, making the measurement of upstream or downstream products an inaccurate way to determine original NO concentrations.^[^ ^11^, ^14^, ^15^, ^18^, ^19^, ^20^ ^]^ Chemiluminescence methods rely on NO‐based chemical reactions to quantify NO concentration, but they require an excess of cofactors to ensure that the reaction luminescence signal reflects NO levels rather than a deficiency of reagents, leading to cost‐related limitations.^[^ ^18^ ^]^ Electrochemical sensors can directly detect NO changes over time, but they are limited in spatial resolution by the distance between the electrodes and the number of probes in the solution.^[^ ^21^ ^]^ Some fluorescent probes, such as quantum dots, can directly detect NO, but many have limited temporal resolution due to photobleaching.^[^ ^16^, ^17^, ^21^, ^22^ ^]^ Functionalized single‐walled carbon nanotubes (SWNT) are an example of fluorescent probes capable of direct NO detection, supported by various theoretical fluorescent mechanisms of action, including redox selectivity, nonradiative energy loss, and steric hindrance.^[^ ^23^, ^24^, ^25^, ^26^, ^27^, ^28^, ^29^, ^30^, ^31^, ^32^ ^]^ SWNT offer exceptional temporal resolution, demonstrated by a rapid response to NO with an adsorption rate constant of 0.001 s^−^^1^ μM^−^^1^, and the resistance to photobleaching, enabling continuous monitoring of NO fluctuations over short time frames and sustained over extended periods.^[^ ^25^ ^]^ The small size of SWNT leads to their internalization by cells, but multiple SWNT immobilization methods have been developed to avoid cellular uptake and allow for the detection of extracellular analytes.^[^ ^27^, ^33^, ^34^, ^35^, ^36^, ^37^, ^38^, ^39^ ^]^ A recently developed SWNT platform for NO sensing, which is based on avidin–biotin interaction, provides even SWNT distribution on a glass surface with a stable fluorescence profile throughout multiple washing steps and over a 10 days period.^[^ ^29^, ^40^, ^41^ ^]^ These facts combine to make SWNT an ideal candidate for in vitro NO testing.

Compared to traditional methods for studying extracellular analyte diffusion, including integrative optical imaging, which tracks intensity changes over time in defined areas,^[^ ^42^, ^43^ ^]^ and microelectrodes, which provide localized analyte readings within the micrometer distance of the electrodes.^[^ ^44^, ^45^, ^46^ ^]^ The uniformly distributed SWNT platform offers superior spatial resolution, each nanosensor functions as an independent probe, providing comprehensive coverage beneath and around cells. The SWNT platforms enable, for the first time, precise pixel‐level mapping of extracellular NO, delivering unprecedented insights into NO dynamics.

**Figure 1:**
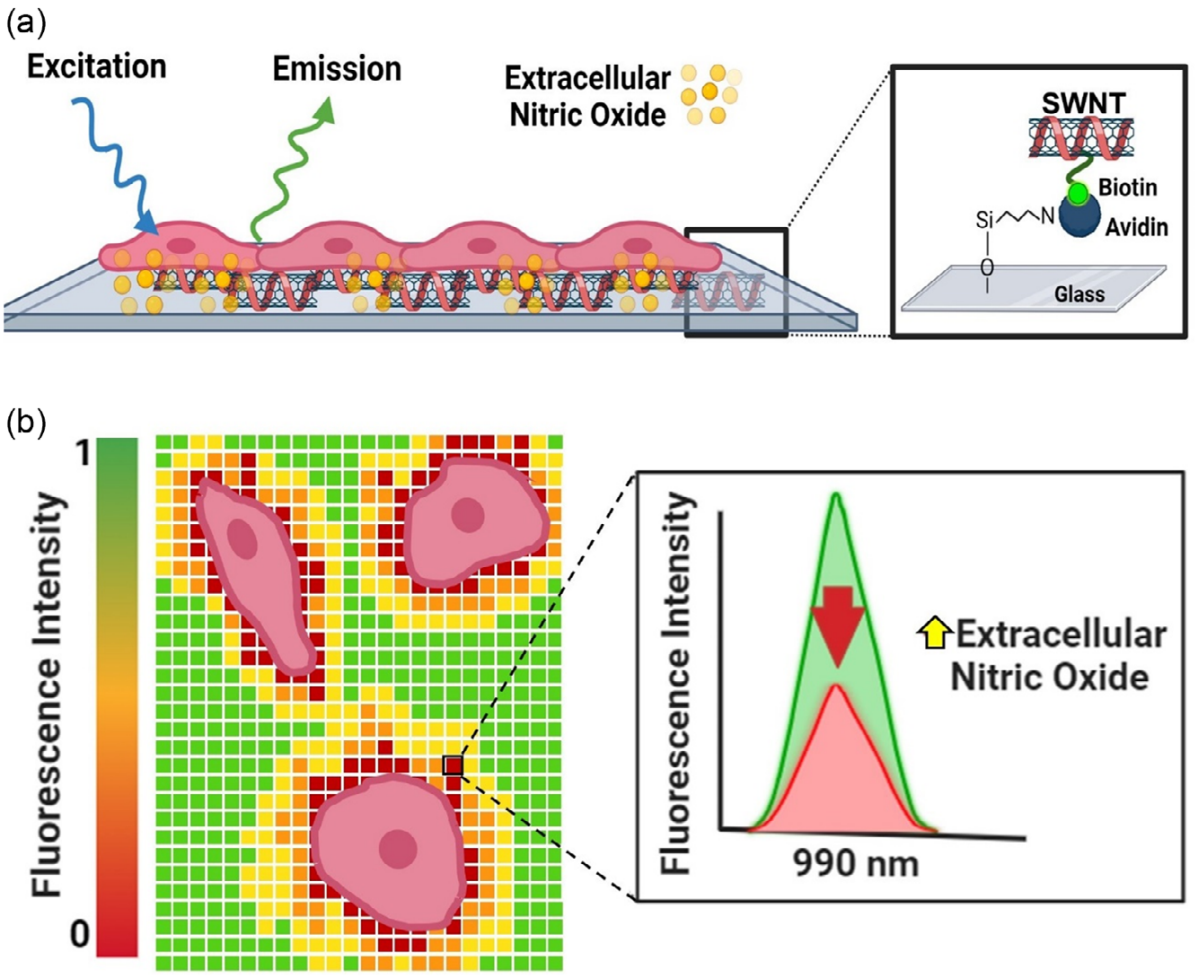
Scheme of the proposed sensing mechanism to detect NO efflux gradients using SWNT platforms. a) SWNT immobilization on glass slide via avidin–biotin interactions. b) Representation of the turn‐off biosensor functionality. The extracellular NO released by the cells decreases SWNT's fluorescence intensity, which is quantified at the pixel‐level.

In this study, we utilize SWNT platforms for spatial and temporal detection of NO efflux produced by two cell lines: human (THP‐1) and murine (RAW 264.7) macrophage cells (**Figure** **1**a). To study NO diffusion gradients from the cell into the extracellular environment, instead of using common image processing algorithms focused on evaluating fluorescence intensity changes over a general geometric area,^[^ ^42^, ^47^, ^48^ ^]^ we developed a MATLAB code to quantify the fluorescent diffusion gradients from cells with various morphologies by generating regions of interest (ROIs): under the cell, first level, and second level of expansion from the cell edge (Figure 1b). The analysis includes cell contour identification using Roboflow, an open web‐based computer vision tool, transferring the cell's coordinates to SWNT fluorescence files, and quantifying fluorescence intensity changes using MATLAB. This spatial‐temporal analysis of extracellular NO represents the first example of quantification of extracellular NO gradients and diffusion with pixel specificity.

## 2 Results and Discussion

### 2.1 SWNT Platform Detects NO Produced by Cells

**Figure 2:**
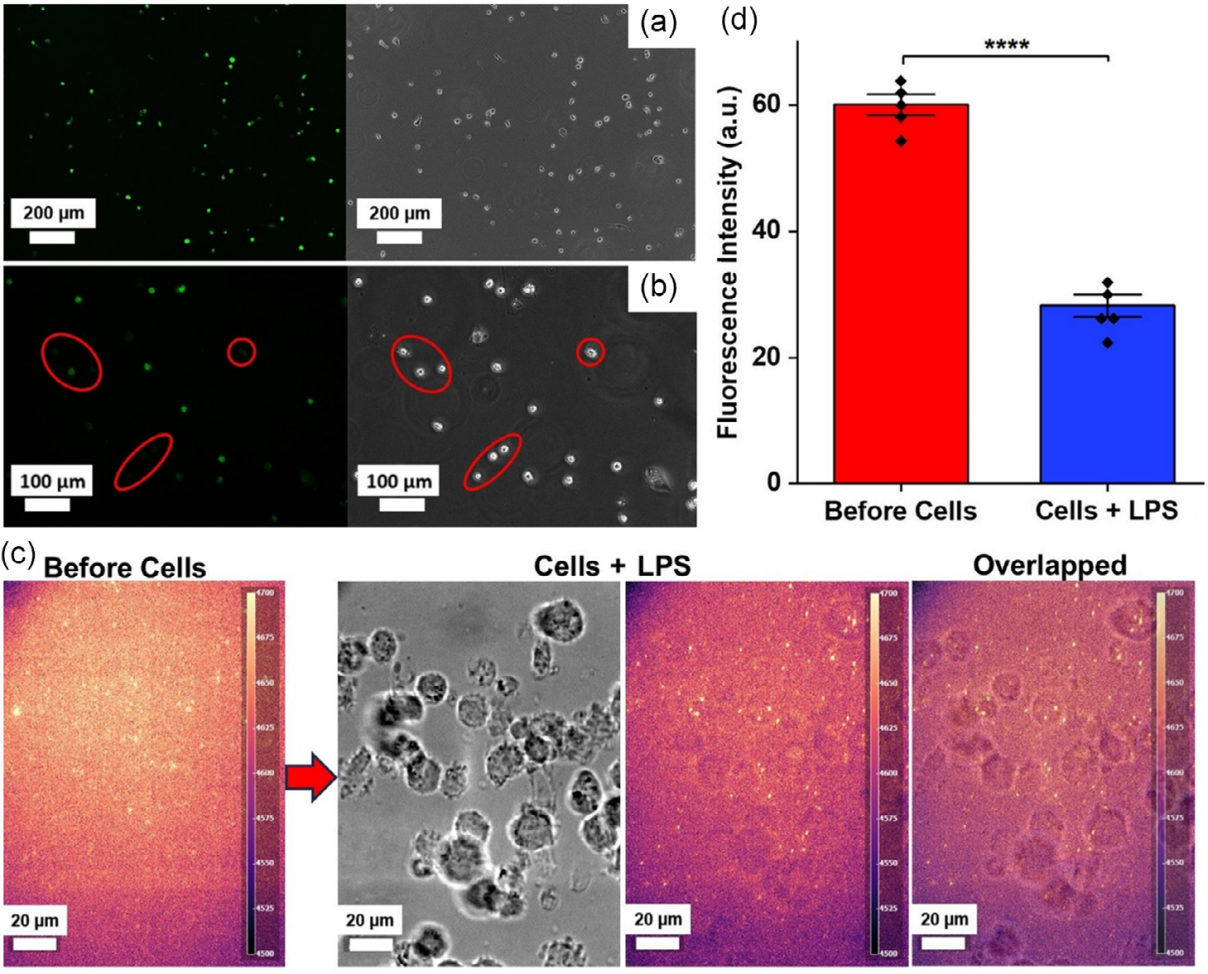
Detection of NO produced by THP‐1 cells using SWNT platforms. a) Intracellular NO production was visualized with the DAF‐FM dye (left) and bright‐field images (right), scale bar: 200 μm. b) NO production varies among cells; red circles show cells with low fluorescence intensity, scale bar: 100 μm. c) Fluorescence heatmaps show the SWNT platform's even fluorescence distribution before seeding the cells and the decrease in the fluorescence intensity under the cells after inducing NO production with LPS. d) Average fluorescence intensity after inducing extracellular NO production (*n* = 3). Values represent the mean ± standard deviation. Statistical analysis was performed using an unpaired *t*‐test. Significant difference: *p* > 0.05 (ns), *p* ≤ 0.0001 (****).

Intracellular NO production by THP‐1 cells was detected through the addition of 4‐amino‐5‐methylamino‐2′,7′‐difluororescein diacetatethese (DAF‐FM DA), a membrane‐permeable dye that emits fluorescence at 515 nm when reacting with NO. After THP‐1 monocytes were differentiated into adhesive macrophages, they were incubated for 24 h with 1 μg mL^−1^ of lipopolysaccharides (LPS), which induce NO production, and were stained with 10 μM DAF‐FM DA for 30 min prior to bright‐field and fluorescent imaging. NO production was observed throughout the cell population, as shown in the representative bright‐field and fluorescence images in **Figure** **2**a,b. Cells displayed distinctively different levels of fluorescence intensity (Figure 2b), indicating diverse NO production among the cells.

NO produced and released by THP‐1 macrophage cells, after being incubated with LPS for 24 h, was assessed by measuring changes in the sterile SWNT platform's fluorescence intensity. The (AT)_15_ wrapped SWNT, turn off NO sensors, show an even fluorescence distribution before cell seeding and localized decreases in fluorescence intensity at cell‐occupied sites (Figure 2c). The colocalization of fluorescence quenching and cell location indicates the ability of SWNT platforms to detect extracellular NO released by THP‐1 cells. The significant change in the average fluorescence intensity before and after inducing the cellular NO production (Figure 2d) indicates the SWNT platform's capability to detect NO produced by the cells.

### 2.2 The Cells are not Affecting the Sensor Functionality

**Figure 3:**
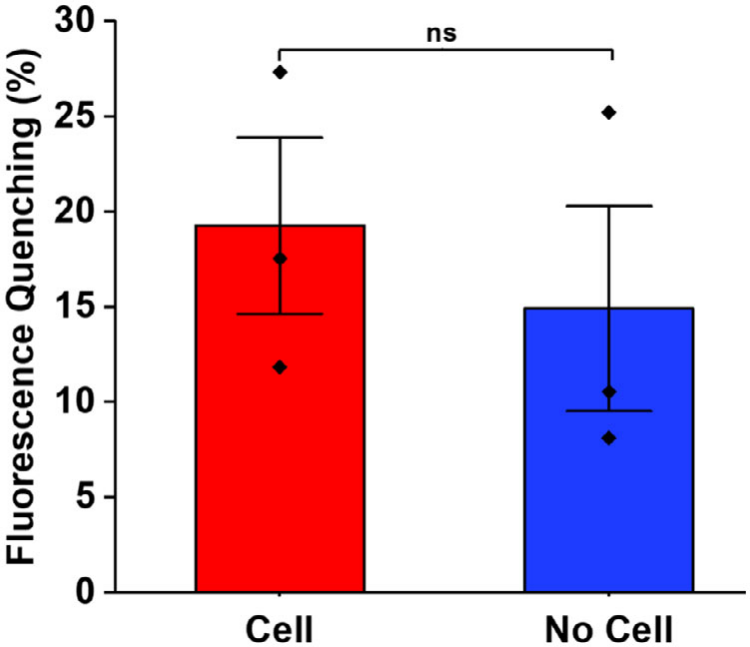
Evaluation of sensor functionality in the presence of cells. Fluorescence quenching was assessed after 30 min incubation with a NO donor in areas with and without cells (*n = *3). Values represent the mean ± standard deviation. Statistical analysis was performed using an unpaired *t*‐test. Significant difference: *p* > 0.05 (ns).

Extracellular matrix proteins have been shown to adsorb onto SWNT surfaces,^[^ ^49^, ^50^ ^]^ so it is important to assess the impact of the cells on the SWNT's functionality. DETAnonoate, a NO donor, was used to assess the SWNT platform's sensing capabilities in the presence of cells. SWNT platforms seeded with THP‐1 macrophage cells, after the 56 h differentiation process into macrophage phenotype, were imaged before and after a 30 min incubation with 100 nM DETAnonoate. Fluorescence quenching in response to NO was calculated for equivalent‐sized regions with and without cells (**Figure** **3**). Although the fluorescence quenching for both regions was comparable after being treated with the same NO concentration, the region under the cell presented a slightly greater quenching percentage, possibly due to additional NO released from the cells. These results indicate that the THP‐1 adhesion network does not impact the functionality of the SWNT platform.

It is important to note that the DETAnonoate release rate and stability depend on the solution or suspension media.^[^ ^51^ ^]^ Due to pH alterations, the NO release profile in PBS is more stable than in complete media.^[^ ^51^ ^]^ Consequently, the same DETAnonoate concentration used in this experiment could result in different degrees of quenching in other conditions.

### 2.3 Temporal Extracellular NO Sensing

**Figure 4:**
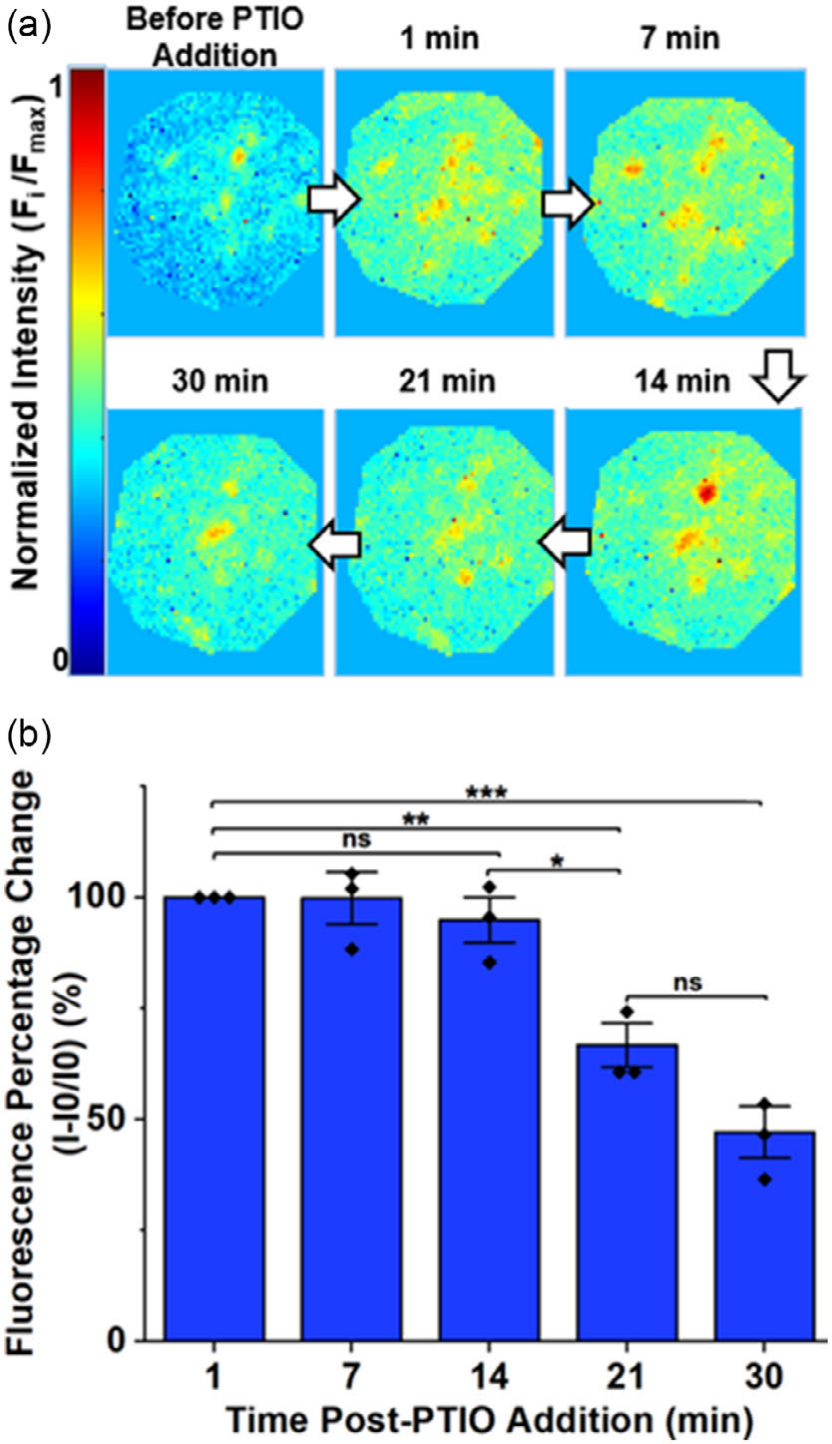
Temporal extracellular NO sensing functionality. a) Area under the cell showing extracellular NO changes over time modulated with PTIO, a NO scavenger. b) Fluorescence quantification changes relative to the initial fluorescence intensity before the PTIO addition for multiple cells, (*n = *3). All values represent the mean ± standard deviation, *p* values were performed by one‐way ANOVA Tukey's post hoc test. Significant difference: *p* > 0.05 (ns), *p* ≤ 0.05 (*), *p* ≤ 0.01 (**), *p* ≤ 0.001 (***).

To evaluate the SWNT platform's response to NO changes over time in the presence of cells, we modulated extracellular NO concentration using 2‐phenyl‐4,4,5,5‐tetramethylimidazoline‐1‐oxyl‐3‐oxide (PTIO), a NO scavenger. THP‐1 macrophages cultured on the SWNT platforms and incubated for 24 h with LPS to induce NO production were treated with 100 μM PTIO for 30 min. Fluorescence heatmaps were acquired before and during the PTIO incubation to monitor changes in extracellular NO levels (**Figure** **4**). Heatmaps of the region under the cell show an initial quenching characteristic of extracellular NO released by the cell, followed by a quick recovery in fluorescence intensity after PTIO addition as a result of its NO scavenging action (Figure 4a). Results from the heatmaps are backed up by quantitative analysis in which the average fluorescence intensity was found for each ROI and compared to the average fluorescence intensity for the other time points. The changes relative to the recovered intensity after PTIO addition were calculated and normalized to percentages (Figure 4b). As can be seen in both Figure 4a,b, the recovered fluorescence intensity results from scavenged extracellular NO and highlights the SWNT platform's fast response to NO changes. The SWNT's response rate, under 1 min, when using 100 μM PTIO, aligns with the reaction rate between PTIO and NO that has been reported in the literature, which estimates an NO half‐life of 2.3–0.3 min in the presence of 30–300 μM PTIO.^[^ ^52^, ^53^ ^]^ The adhesion of cells to the SWNT platform does not appear to delay the SWNT's sensing response rate.

Between 1‐ and 14‐min post‐PTIO addition, the fluorescence intensity of the SWNT platform remains high, but by the 21‐min mark, the fluorescence starts returning to the pre‐PTIO value. This fluorescence decrease is attributed to either a decrease in PTIO activity or increased NO production from the macrophage cells in response to the PTIO.

### 2.4 SWNT Platform Demonstrates Spatial Extracellular NO Sensing

**Figure 5:**
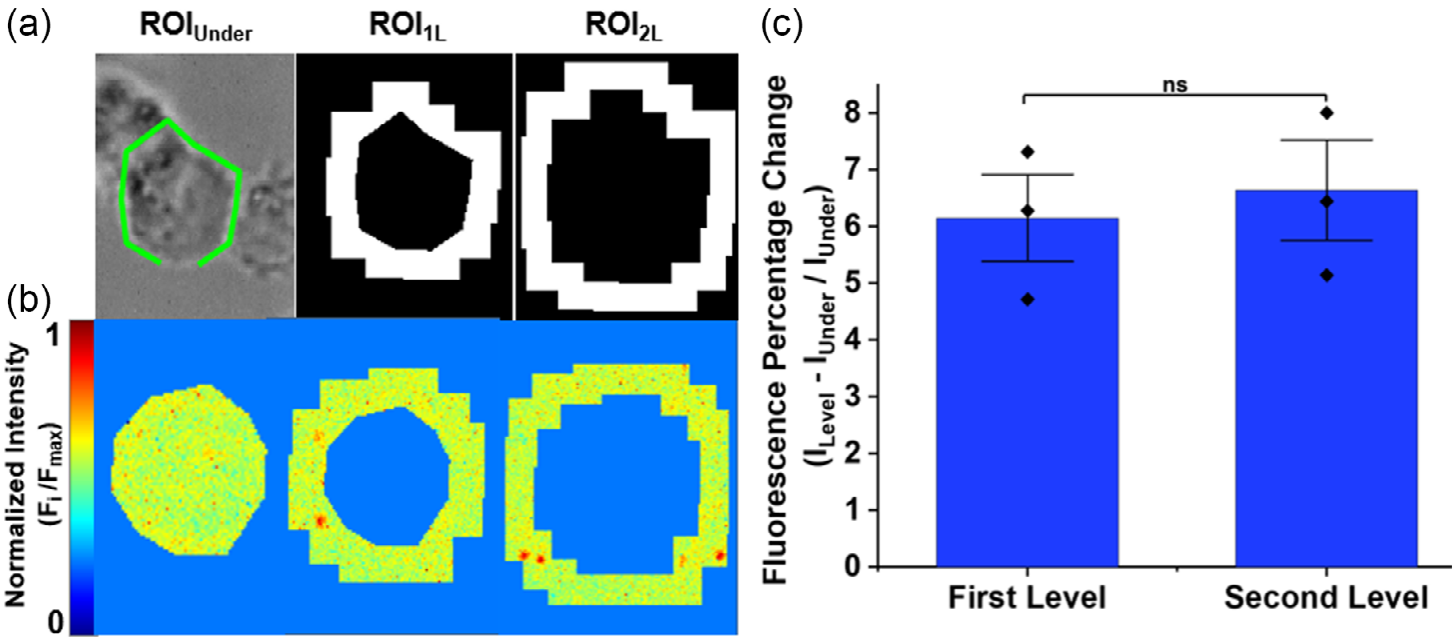
Spatial sensor functionality to detect NO efflux gradients. a) Mask depicting the regions of study: under the cell (ROI_Under_), the first level of expansion from the cell edge (ROI_1L_), and the second level of expansion from the first level (ROI_2L_). b) Fluorescence heatmaps for the ROIs. c) Quantification of fluorescence percentage change across the different ROI (*n = *3). All the values represent the mean ± standard deviation, *p* values were performed by one‐way ANOVA Tukey's post hoc test. Significant difference: *p* > 0.05 (ns).

NO is a paracrine signaling molecule that diffuses into the extracellular environment to induce changes in neighboring cells.^[^ ^54^ ^]^ To study extracellular NO release gradients, the NO production and release from THP‐1 macrophage cells growing on SWNT platforms, was induced with a 24 h LPS incubation period, and a computer vision system was used to analyze the fluorescence intensity in specific ROIs: ROI_Under_, representing NO release under the cell; ROI_1L_, representing the first NO diffusion level from the cell edge; and ROI_2L_, representing a second expansion level from ROI_1L_. Cell contours were delimited using Roboflow segmentation tools, and the coordinates were exported to MATLAB to generate binary masks (ROIs) using pixel connectivity and dilation functions (**Figure** **5**a and Figure S1a, Supporting Information). The ROI masks were applied to the raw SWNT fluorescence data to segment and independently analyze the extracellular NO levels for each region (Figure 5b). Considering the cell as the source of NO release, we expected lower fluorescence intensity under the cell and a gradient increase in intensity as NO diffuses from the cell edge. Figure 5c represents the quantification of fluorescence intensity between expansion levels in relation to the region under the cell. ROI_1L_ and ROI_2L_ showed a significant increase in fluorescence intensity of ≈6% compared to ROI_Under_, representing an increase in NO when moving from under the cell to the area around the cell. There was no significant difference between ROI_1L_ and ROI_2L_, indicating that the NO concentration at both distances from the cell was similar. More ROIs could be studied in the future to see if the NO concentration decrease continues to change with additional distance from the cell, but this needs to be performed in a more controlled environment. As highlighted in Figure 2b, different cells have different concentrations of NO, these differences could result from association with surrounding cells or differences in cell cycle. Analysis of multiple cells that are at similar distances from other cells and at similar cell cycle stages will be performed in the future to elucidate more nuanced information about NO released by cells, but these initial experiments are the first step in demonstrating quantification of NO gradients.

### 2.5 Detecting Directionality of Cellular NO Efflux

**Figure 6:**
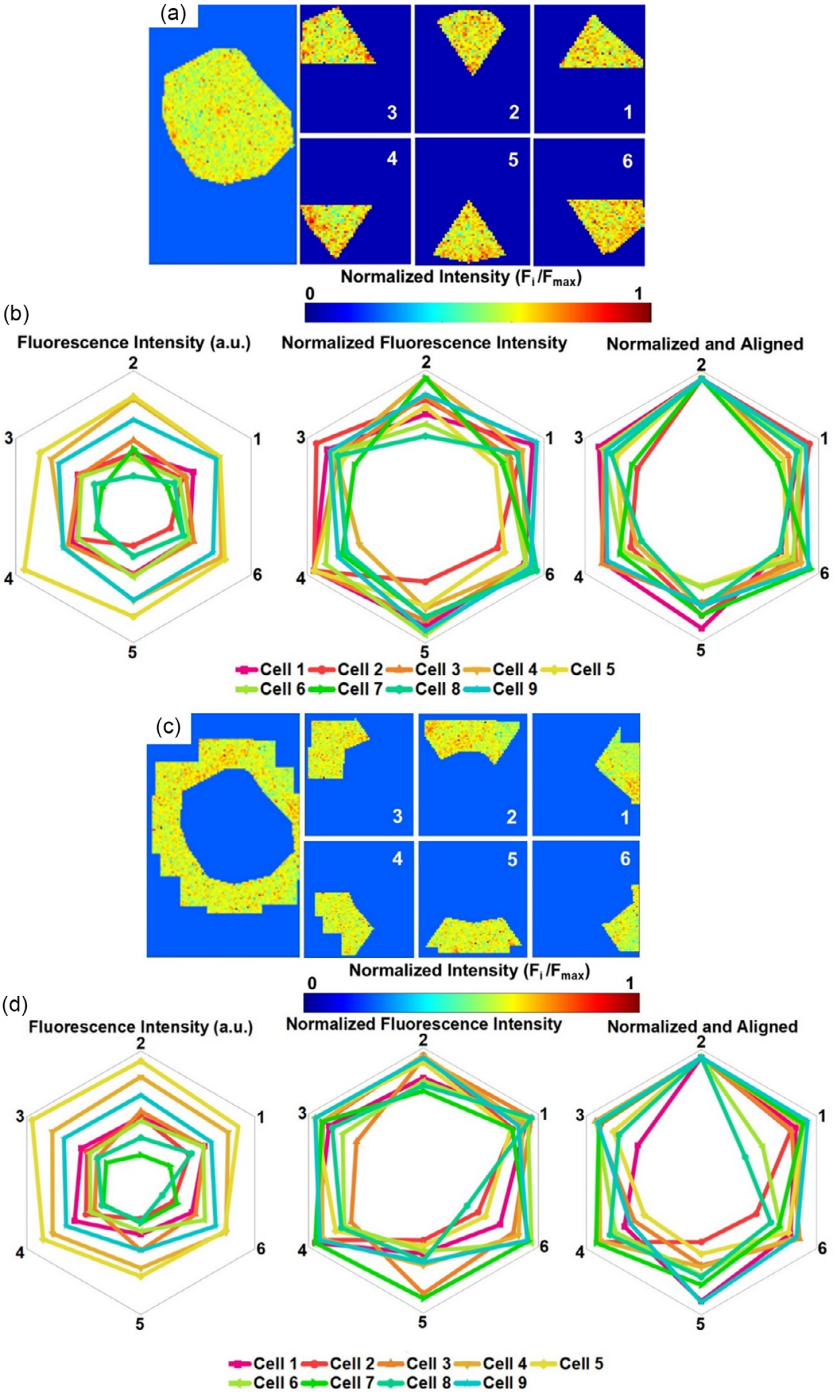
Extracellular NO efflux directionality. a,c) The area under and around the cell is divided into six equal sections. b,d) The average fluorescence for the segments is represented as hexagon vertices. The fluorescence intensity is represented as an axial line with the minimum value at the middle of the plot, and the maximum intensity value at the hexagon border, and multiple cells are represented with different colors. The data are also normalized so that the largest fluorescent value is represented equally between cells and then realigned so that the largest value is at the 2‐segmentation location.

To determine whether NO is released homogeneously under and surrounding cells, each ROI was divided into six segments, each spanning 60°: segment 1 covers the region from 0 to 60°, segment 2 from 60 to 120°, segment 3 from 120 to 180°, and so on, up to 360° (**Figure** **6**a,c). The average fluorescence intensities for each segment under (Figure 6b) and surrounding the cells (Figure 6d) were illustrated as hexagon vertices, with fluorescence intensity represented as a radial line with the minimum value at the center of the plot, and the maximum value at the edge of the hexagon. The variation in fluorescence intensity among cell segments indicates that cellular NO efflux is not equally released under or around the cells. Since the point of 0° was arbitrarily assigned to the cells related to an x–y grid that the cells are unaware of, we looked at the data in two additional formats, first the raw fluorescence intensity was plotted after normalization to a maximum value of 1 and then plotted after normalization and alignment of the maximum values. The differences seen in the realigned data indicate that not only is NO concentration different between cells, but the location of the NO within and around the cells varies. The localized extracellular NO release could be due to the location of each cell relative to other cells on the platform, the location of organelles within the cell, or multiple other differences between the cells.^[^ ^55^ ^]^ Future research will be undertaken in which we try to control for these cell‐to‐cell interactions and can elucidate the factors driving the differences that are observed.

### 2.6 Extracellular NO Detection in Multiple Cells

**Figure 7:**
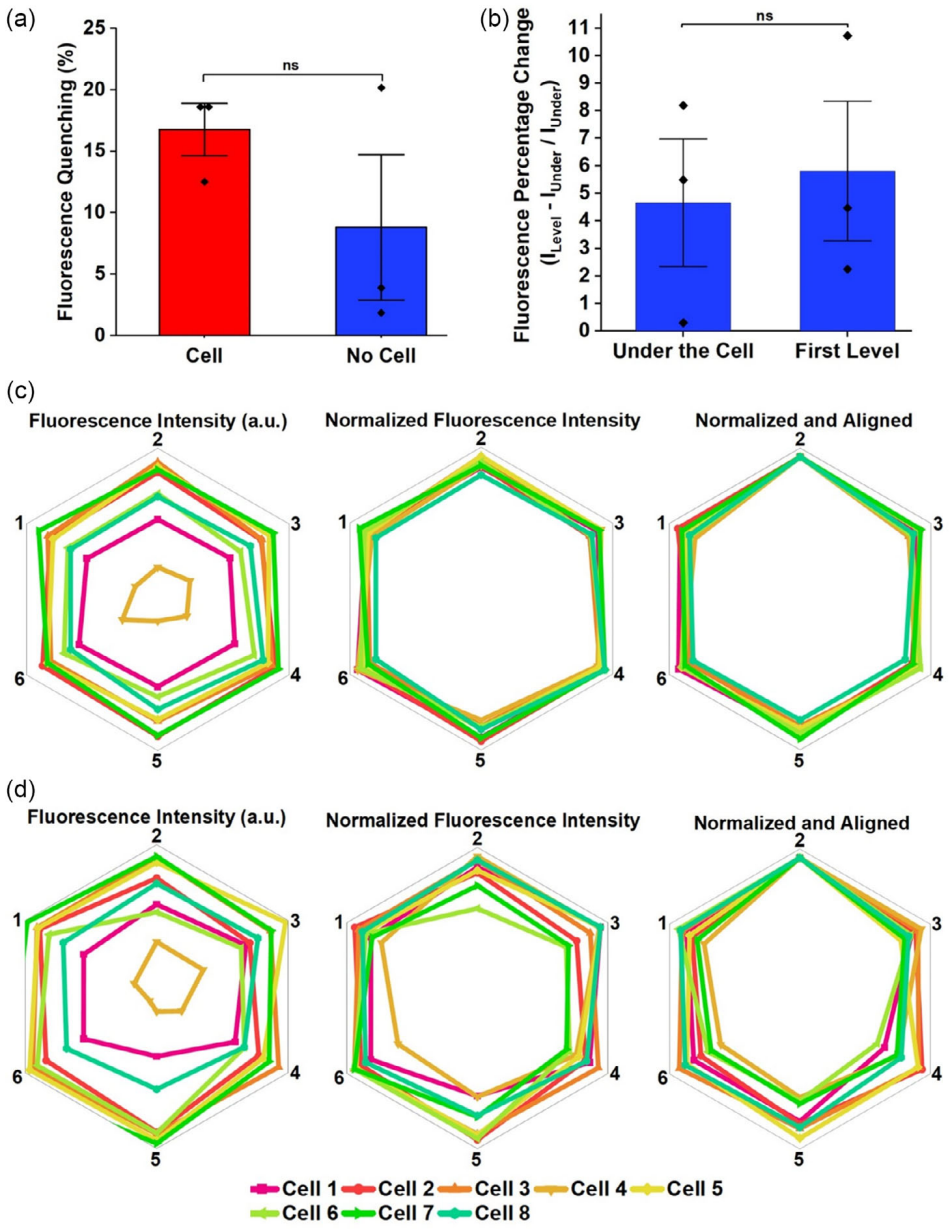
Extracellular NO detection in murine macrophage. a) RAW 264.7 cell adhesion does not affect the SWNT platform's turn‐off response to NO. b) Average fluorescence percentage change with respect to the region under the cell (*n* = 3). All the values represent the mean ± standard deviation. Statistical analysis was performed using an unpaired *t*‐test. Significant difference: *p* > 0.05 (ns). c) Average fluorescence intensity illustrating spatial NO efflux directionality under the cell. d) Average fluorescence intensity at a level of expansion.

To demonstrate the universal applicability of the methods that we developed, the SWNT platform's extracellular NO detection was also evaluated with the murine macrophage cell line RAW 264.7.^[^ ^56^ ^]^ The RAW 264.7 cells naturally attach to surfaces through specific adhesion proteins,^[^ ^50^, ^56^, ^57^ ^]^ but tests showed that they did not impact the SWNT's sensing capabilities (**Figure** **7**a). The results were consistent with those observed for THP‐1 cells: the decrease in fluorescence intensity after DETAnonoate incubation was not significantly different for areas with and without cells, although the percent quenching under the cells was higher than in the regions without cells.

The NO diffusion gradients were also tested for the RAW 264.7 cells and once again the method developed was able to quantify fluorescent intensity under and surrounding the cells (Figure 7b–d). Interestingly, except for one of the cells, arbitrarily labeled cell 4, the NO release under and surrounding the RAW 264.7 cells appeared more uniform than THP‐1 cells. This finding hints at differences in how different cells release NO and will be further studied in the future. However, the most important finding is that the SWNT platform and the method developed to analyze NO gradients continue to work for additional cell types.

## 3 Conclusion

The SWNT platform provides NO efflux gradient information with pixel‐level resolution for multiple cell types, making it the first of its kind for biological applications. Our results demonstrate that the SWNT platform provided a uniform fluorescence sensory array, enabling the determination of directionality and variations in extracellular NO released under and around the cells. We also demonstrated that cellular adhesion does not impact the SWNT platform's response to NO over time. Overall, the exceptional time and spatial resolution of the SWNT platform enable the study of extracellular NO in cellular communication, enhancing our understanding of physiological and pathological processes for more accurate diagnostic and therapeutic approaches.

## 4 Experimental Section

4.1

4.1.1

##### SWNT Functionalization

SWNT were dispersed with DNA oligos according to methods previously described.^[^ ^32^, ^58^ ^]^ Briefly, CoMoCAT 6,5 SWNT (Sigma) were suspended with single‐stranded DNA in a 2:1 mass ratio in nanopure water. The DNA oligonucleotides used were a 1:1 volumetric ratio of (AT)_15_ and 5′‐biotinylated (AT)_15_ oligos (Integrated DNA Technologies). To promote DNA functionalization, the suspension was initially bath‐sonicated, followed by ultrasonication to unbundle and disperse the individual nanotubes. The solution was then centrifuged for 180 min at 16100 relative centrifugal force to remove any unwrapped SWNT from the supernatant.

##### Fabrication of Biocompatible SWNT Platform

SWNT immobilization onto glass slides followed our previously published protocols.^[^ ^29^, ^40^, ^41^ ^]^ Briefly, a 5‐step chemical technique was used to treat glass slides to create an avidin surface that interacted with the biotin from the SWNT. The first step consisted of the immersion of glass slides in a piranha solution (70% H_2_SO_4_, 30% H_2_O_2_), followed by a solution of 3‐Glycidyloxypropyl trimethoxysilane (3‐GPTMS) in 98% ethanol for an entire night to sterilize them. To preserve the sterility of the slides in nonsterile steps including oven incubation and the addition of amine groups for further avidin immobilization, specialized boxes to transport the slides to the biosafety cabinet were used. Prior to use, the solutions used in stages three and four were sterilized.

##### Cell Culture

The murine macrophage line RAW 264.7 (ATCC TIB‐71) and the human monocyte cell line THP‐1 (ATCC TIB‐202) were incubated at 37 °C/5% CO_2_ and cultured in medium supplemented with 10% fetal bovine serum (FBS). RAW 264.7 cells were maintained in Dulbecco's Modified Eagle Medium (DMEM), while THP‐1 cells were cultured in Roswell Park Memorial Institute (RPMI) 1640 medium. To differentiate the monocytes into the adherent macrophage‐like phenotype, THP‐1 cells were treated with phorbol 12‐myristate‐13‐acetate (PMA, Sigma P1585) (16 ng mL^−1^) in RPMI complete medium for 16 h, followed by 56 hr incubation with complete media before the start of experiments.

NO production was induced by preincubating the cells with lipopolysaccharides from *Escherichia coli* (LPS, Sigma‐Aldrich L5418) (1 μg mL^−1^) for 24 h.

##### Nitric Oxide Modulation

To scavenge NO from the system, the NO scavenger 2‐phenyl‐4,4,5,5‐tetramethylimidazoline‐1‐oxyl‐3‐oxide potassium salt (PTIO, Cayman 81540) was prepared as a stock of 1 mM in DMSO. A final concentration of 100 μM PTIO in media was used on the SWNT platforms.

##### Intracellular Nitric Oxide Assay

Intracellular NO production was assessed using 4‐Amino‐5‐Methylamino‐2′,7′‐Difluorofluorescein Diacetate (DAF‐FM Diacetate, Thermofisher D23844) according to the manufacturer's protocol. Cells were stained with DAF‐FM Diacetate (5 μg mL^−1^) and incubated for 40 min at 37 °C/5% CO_2_. Then, the cells were washed two times with 2 mL of water and incubated for an additional 30 min in fresh media. Fluorescence images were captured using a Keyence inverted fluorescent microscope with emission at 515 nm.

##### Extracellular Nitric Oxide Fluorescence Imaging and Microscopy

RAW 264.7 and THP‐1 cells were seeded and differentiated on the SWNT platform. After the adherent macrophage phenotype was confirmed, NO production was stimulated by incubating the cells with LPS for 24 h. Fluorescence intensity measurements of the SWNT platforms in response to extracellular NO were conducted using a custom‐built near‐infrared microscope (Photon etc.). The SWNT platforms were excited with a 561 nm laser, and 990 nm pixel‐by‐pixel emission intensity information was captured using an InGaAs camera (Xenics, Xeva‐1.7‐320 TE3). The h5 files and bright‐field images have a pixel size of 0.2646 μm, with an image resolution of 512 × 640 at 96 dpi, and were further analyzed using PhySpec software and MATLAB.

##### MATLAB—Extracellular NO Data Analysis

We delimited the cell edge using the intelligent polygon labeling tool in the software Roboflow. The cell contour coordinates format was adjusted with Microsoft Office and exported to MATLAB to generate a binary mask defining the ROI under the cell. MATLAB's pixel dilation functions were applied to each coordinate point on the cell contour to generate the expansion levels from the cell edge. Loops adjusted the thickness of the expansion area to ensure an even degree of expansion with amorphous cell border regions. To quantify NO diffusion gradients as a function of the distance from the cell edge, we created a secondary level of expansion similar to the first one. The different ROI, under, first, and second levels of expansion, were analyzed independently by subtracting one from the other using heatmaps and average fluorescence intensity. An element‐wise multiplication of the respective ROI with the raw fluorescence values from the h5 files was performed for quantitative and qualitative analysis of each segment. The detailed process is described in the Supporting Information.

To analyze the directionality of the NO produced by the cell, we divided the area under the cell and the expanded level into six segments. The MATLAB centroid function identified the center of the cell mask and was used as a reference to divide the different ROI into segments of 60° angles.

To compare NO concentration at different time points following exposure to PTIO, we quantified the average fluorescence intensity within the region under the cell for each fluorescence image at the respective time points: before PTIO addition and at 1, 7, 14, 21, and 30 min postaddition. The fluorescence percentage change was calculated relative to the initial quenched fluorescence. Since cells are producing NO at different proportions, the initial fluorescence quenching reading was different between cells. To evaluate/compare multiple cells, we normalized the readings by making 100% the maximum, initial percentage change after the PTIO addition.

To compare NO concentration in different ROIs, we followed a similar protocol as that performed for the different time points.

## Conflict of Interest

The authors declare no conflict of interest.

## Author Contributions

**Ivon Acosta Ramirez**: data curation (lead); formal analysis (lead); investigation (lead); methodology (lead); project administration (lead); software (lead); supervision (lead); validation (lead); visualization (lead); writing—original draft (lead); writing—review and editing (equal). **Sruti Das Choudhury**: conceptualization (equal); data curation (equal); software (lead); supervision (equal); validation (equal). **Carley Conover**: investigation (supporting); methodology (supporting); visualization (supporting); writing—original draft (supporting). **Omer Sadak**: conceptualization (supporting); investigation (supporting); methodology (supporting). **Nicole M. Iverson**: conceptualization (lead); funding acquisition (lead); project administration (lead); resources (lead); supervision (lead); visualization (equal); writing—original draft (equal).

## Supporting information

Supplementary Material

## Data Availability

The data that support the findings of this study are available from the corresponding author upon reasonable request.
