# Supplementary material for: Programming‐Assisted Imaging of Cellular Nitric Oxide Efflux Gradients and Directionality via Carbon Nanotube Sensors
Source: Small Sci. 2025 Feb 4;5(4):2400493. doi: 10.1002/smsc.202400493 (PMC12245041; doi:10.1002/smsc.202400493)
Supplement: Supplementary file 1 — Supplementary Material [file SMSC-5-2400493-s001.pdf]

Programming-Assisted Imaging of Cellular Nitric Oxide Efflux Gradients and Directionality Via Carbon Nanotube Sensors

Ivon Acosta Ramirez, S Das Choudhury, Carley Conover, Omer Sadak, and Nicole M Iverson.*

I. Acosta Ramirez, C. Conover, O. Sadak, N.M. Iverson

Department of Biological Systems Engineering

College of Agricultural Sciences and Natural Resources

University of Nebraska-Lincoln

Lincoln, Nebraska, 68504, United States.

E-mail: iverson@unl.edu

S. Das Choudhury

School of Natural Resources

College of Agricultural Sciences and Natural Resources

University of Nebraska-Lincoln

Lincoln, Nebraska, 68504, United States.

Supplemental information:

Image processing method

1. Cell contour identification.

To improve the precision of analyzing cells with diverse morphologies, including rounded and elongated shapes, we utilized Roboflow for its instant segmentation and automated cell contour identification capabilities. This tool delineates cell boundaries as polygons using multiple points or coordinates, enabling accurate identification of complex cell morphologies.

Roboflow detects precise contours even in overlapping cells **(Figure S1a),** making it particularly valuable for heterogeneous cell populations.

After the initial automated detection, the software allows for manual adjustments, such as refining the boundary locations or adding additional delimiting points, to ensure an accurate definition of irregular cell shapes as seen in **Figure S1a.** Once the cell contour is identified, Roboflow provides the cell central (x,y) coordinates for recognition purposes, along with the respective list of all the respective delimiting (x,y) coordinates (**Figure S1b**). This coordinate data is subsequently exported and formatted for integration into a MATLAB program to define regions of interest for further analysis.

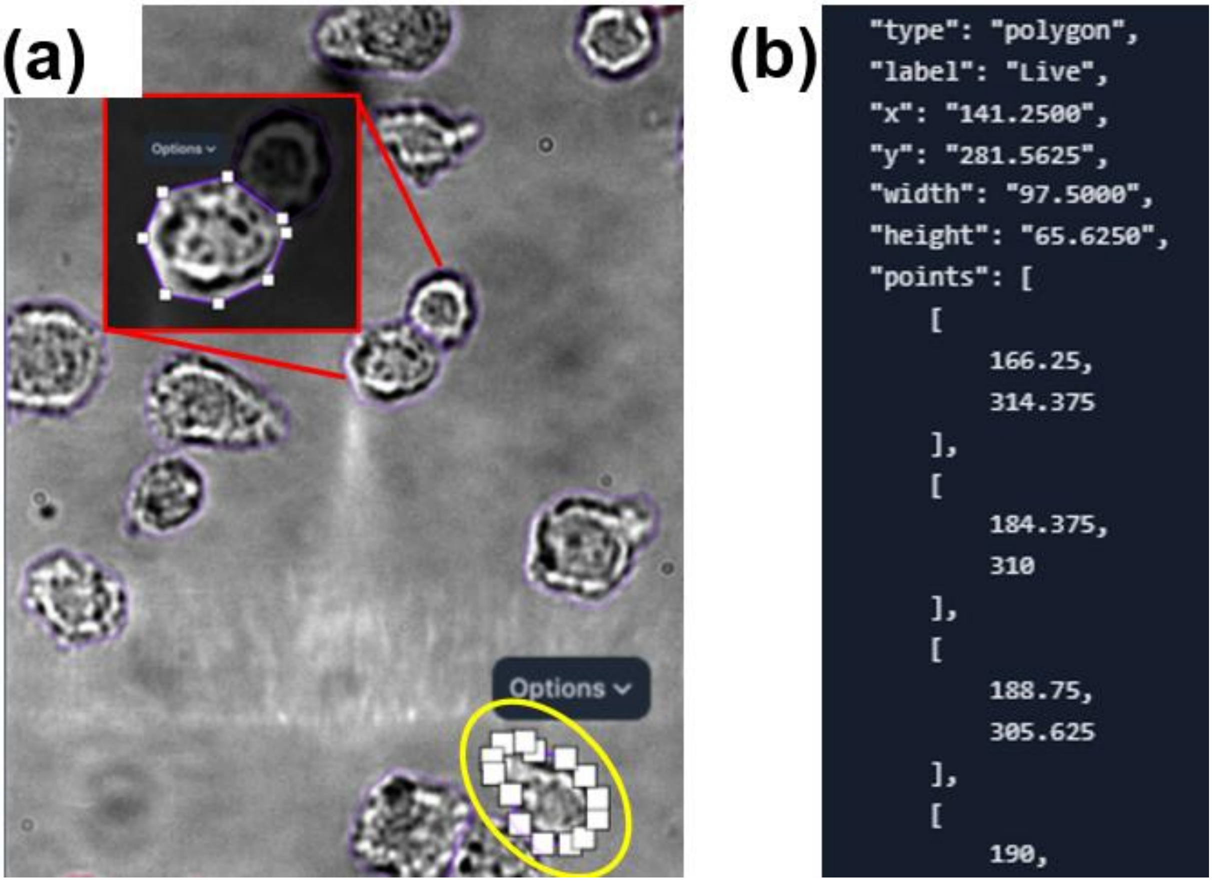

Figure S1. Roboflow functionality for automatic cell contour segmentation. (a) Individual cell contours are automatically identified and outlined with purple lines. The segmentation accurately detects diverse cell morphologies, highlighted in yellow, and precisely resolves overlapping cell contours, as demonstrated in the red zoomed-in region. (b) Roboflow exports the segmented cell contour coordinates, along with the corresponding (x, y) central coordinates for each cell, enabling precise tracking and analysis.

2. *Regions of Interest (ROI) definition for fluorescence intensity diffusion analysis*

Regions of interest (ROI) were defined to quantify fluorescence intensity beneath the cell and to analyze the diffusion of nitric oxide (NO) from the cell edge into the extracellular space. Binary masks were created and multiplied by the corresponding SWNT fluorescence data in MATLAB for this purpose. The binary mask for the region beneath the cell (ROI_{Under}) generated using the cell contour coordinates obtained from Roboflow. Within the defined cell contour, pixel values were set to one, while all other pixel values were set to zero.

To quantify NO diffusion gradients beyond the cell boundary, two successive expansion levels were created: the first level of expansion (ROI_{1L}) immediately adjacent to the cell edge, and the second level of expansion (ROI_{2L}), extending outward from $ROI_{1L.}$ ROI_{1L} was generated using MATLAB's dilation function, imdilate, with the structural element strel set to a square shape of diameter 5. This process added four additional pixels to the binary mask around each of the original cell boundary pixels **(Figure S2a)**. A for-loop controlled the iterative dilation process to achieve the desired thickness. Since the dilation expanded uniformly in all directions, including regions already part of ROI_{Under}, any overlapping areas were subtracted from the expanded mask to ensure distinct analysis of the expanded level.

ROI_{2L} was created using a similar approach, starting the dilation process from the outer boundary of ROI_{1L}. Due to the proximity of the original cell boundary coordinates, the expanded levels occasionally overlapped. To address this, we validated the expanded binary masks, ensuring all overlapping pixel values were reduced to one to maintain binary integrity. This step ensured precise delineation of each ROI for accurate fluorescence intensity and diffusion gradient quantification **(Figure S2b)**.

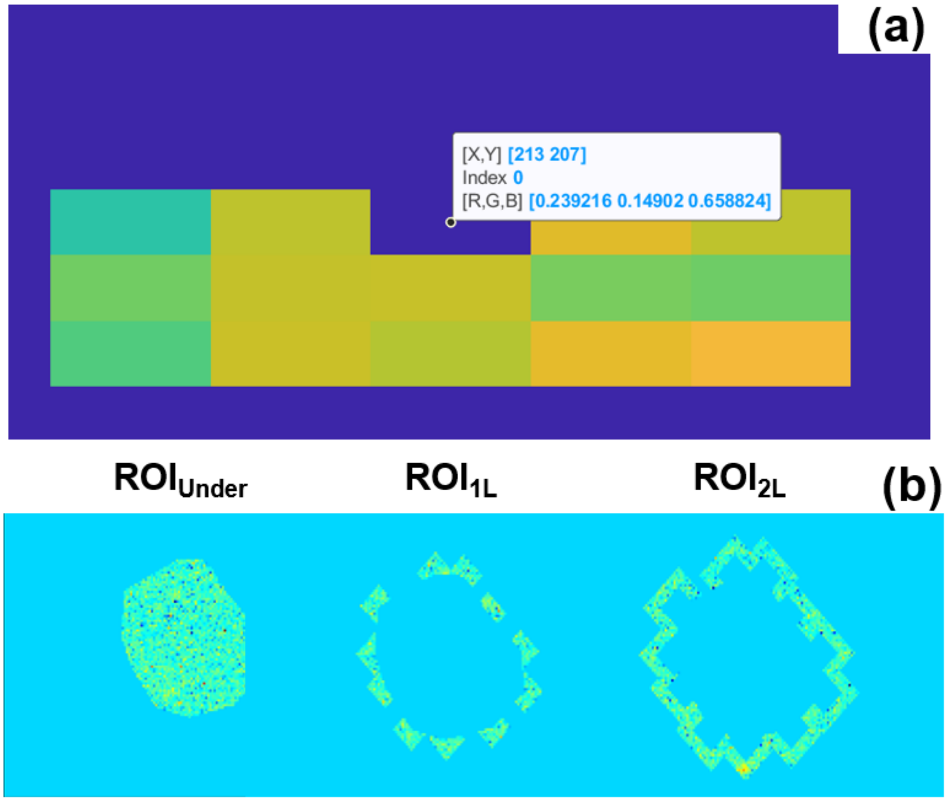

Figure S2. Generation of ROIs from initial cell contour coordinates obtained from Roboflow. (a) Representation of the imdilate(strel) function with a square structural element of diameter 5 applied to a single pixel from the cell contour coordinates. The dilation process expands the selected pixels, merging them into a single binary mask. (b) Illustration of the resulting binary ROI masks after multiplication with the corresponding fluorescence data, demonstrating distinct regions for analysis.
